# A Retrospective Survey of Buprenorphine Substitute Treatment With Minimal Dosage in Heroin Use Disorder

**DOI:** 10.3389/fpsyt.2019.00888

**Published:** 2019-12-04

**Authors:** Wenwen Shen, Qing Wang, Jianbin Zhang, Wenkai Ping, Jiawen Zhang, Weiting Ye, Qianyu Hu, Deniz Cerci, Wenhua Zhou

**Affiliations:** ^1^Ningbo Addiction Research and Treatment Center, Zhejiang Provincial Key Lab. of Addiction, Ningbo University School of Medicine, Ningbo, China; ^2^Ningbo University School of Medicine, Ningbo, China; ^3^Vivantes Wenckebach-Klinikum, Klinik für Psychiatrie, Psychotherapie und Psychosomatik, Berlin, Germany

**Keywords:** buprenorphine, detoxification, maintenance treatment, minimal dosage, heroin use disorder

## Abstract

**Objectives:** It is widely accepted that buprenorphine maintenance treatment (BMT) with dosages above 8 mg daily is effective for patients with heroin use disorder. In this study, the authors evaluated the effectiveness of long-term BMT for heroin users in China, with dosages kept on a much smaller level.

**Methods:** This is a retrospective observational study of 72 patients who had undergone detoxification and continued with buprenorphine maintenance between 2007 and 2016. Measurements such as self-reported relapse status, buprenorphine doses, protracted symptoms, general health condition, and self-reported side effects were included.

**Results:** At the time of interview, 51 patients had remained abstinent at follow-up (including 13 who were opioid-free). The dosages of buprenorphine were 1.33 ± 0.88 (ranging 0.3–3.5) mg/day when maintenance treatment was initiated and 1.2 ± 0.8 (ranging 0.2–3.2) mg/day at the last follow-up. The remaining patients had either relapsed on heroin (n = 11) or switched to compulsory treatment (n=10). In general, abstinent patients had minimal protracted symptoms, especially in physical symptoms. Opioid-free abstainers were more likely to report good physical health than patients on buprenorphine. Predictors of worse outcomes (relapsed or switched to compulsory treatment) were lower education levels, younger age, and younger onset of illicit drug use.

**Conclusions:** This study shows promising results of minimal-dosage BMT in treating heroin use disorder. We recommend further studies applying minimal-dosage BMT in China and worldwide.

## Introduction

Heroin use disorder is a chronic, relapsing disorder characterized by a compulsion to seek and take opioids. It has been linked to the dysregulation of brain regions that mediate reward and stress response. Discontinuation of heroin use causes a negative emotional state, which implies key motivational elements to relapse, such as chronic irritability, emotional pain, malaise, dysphoria, and loss of motivation for natural rewards that drives drug seeking through negative reinforcement mechanisms (1).

Opioid maintenance treatment is increasingly recognized as an effective management strategy in the treatment of heroin use disorders (2). Under supervised agonist delivery, harmful illicit use of drugs can be significantly reduced, and the user’s mental health can be improved (3, 4). A variety of opiate agonists, such as methadone (3), buprenorphine (5), and diamorphine (6), are considered effective as maintenance medication when used under close supervision. Unlike full agonists such as methadone and diamorphine, buprenorphine is regarded as a partial agonist of mu-opioid receptors. At lower doses, it suppresses withdrawal symptoms in abstinent subjects, and as the dose increases, it exhibits a ceiling effect (7). Buprenorphine manifests antagonistic features when it is used with agonists like morphine (8). It may therefore prevent illicit drug use during maintenance and reduce the possibility of buprenorphine misuse.

A sufficient dose to dissolve heroin craving is the key element to the success of opioid maintenance (9, 10). Methadone is often used in dosages around 50–100 mg daily, and buprenorphine in dosages above 8 mg/day is effective in reducing heroin craving (11). An insufficient dose of methadone may lead to illicit drug use (12, 13), or the patient may drop out of treatment (5, 14). However, higher doses than needed may not be helpful for patients. Although one study suggested that additional methadone reduces mood distress (15), another study showed that it may evoke craving for heroin (16). A subtle difference between the two studies lies in the subjective perception of methadone. Subjects in the former study could tell methadone from placebo (15), while those in the latter one could not (16). It indicates that proper dosage of an opioid, neither too much nor too little, is key factor to achieve maintenance efficacy. Correspondingly, in a study using heroin self-administrating rats, we found previously that higher doses may not be necessary to benefit (17). After 1 month’s abstinence, a very low dose of heroin inhibits heroin seeking behavior induced by context or conditioned cues previously associated with heroin reward, contrary to both placebo and higher dosage of heroin (17). This preclinical study also suggests that the needed dose to neutralize craving can be greatly reduced after a withdrawal procedure.

When the concept of buprenorphine maintenance treatment (BMT) was introduced in China in 2006, Ningbo Addiction Research and Treatment Center was among the first to offer the treatment to patients following hospitalization. The clinicians gradually developed minimal-dosage BMT facing the economic realities of the patients. It is hypothesized that once the need and tolerance of an opioid is reduced, much smaller doses of BMT are sufficient to reduce protracted withdrawal symptoms and craving, and regain psychological well-being. As a first step to confirm the hypothesis, we investigate the status of those patients who had received BMT from the Center since 2006.

## Methods

Treatment-seeking heroin-using patients at Ningbo Addiction Research and Treatment Center regularly received detoxification therapy as described in a previous study (18), and later continued with BMT before discharge. Buprenorphine maintenance was carried out in the clinic of the same facility. Buprenorphine hydrochloride sublingual tablets 0.5 mg*10 were manufactured by TIPR Pharmaceutical Responsible Co., Ltd, Tianjin, China. Dosages of buprenorphine are presented as an average per month.

A retrospective study designed to review the outcome of the patients was carried out from March until June 2016 at Ningbo Addiction and Treatment Center. The study was approved by the Ethics Committee of Human Studies of Ningbo Addiction Research and Treatment Center (2016-01).

Inclusion criteria were as follows: 1) Patients were diagnosed with heroin use disorder or heroin dependence prior to receiving treatment. 2) They received a course of detoxification treatment between 2007 and 2016, and were switched to BMT before discharge. 3) The time interval between discharge and follow-up interview was at least 6 months. 4) Patients were maintained on BMT for at least 1 month. 5) Patients or their family members had given oral informed consent to the interview. Exclusion criteria were those who failed to maintain on BMT for 1 month, those who could not be reached, and those who refused to participate.

Contact was made by phone. Face-to-face interviews were carried out in a structured format, which covered perceived side effects, protracted withdrawal symptoms in the last week, and general health conditions at present. Patients who had relapsed were not interviewed, because arranging for follow-up under these circumstances would have been difficult.

### Outcome Indices

The main outcome index was an individual’s relapse. It was recorded as either abstinence or relapse. There were two conditions in people who had been abstinent: Some had quit BMT for some time and were self-reported as drug-free. The rest of the patients had been maintained on buprenorphine and self-reported no use of illicit drugs. Three statuses of patients were coded as relapsed, back on illicit drugs, transferred to compulsory isolated detoxification, and transferred to government-sponsored methadone maintenance therapy (MMT). The reason to code the transfer to compulsory treatments and MMT as relapsed was that it usually happened when a violation of drug regulations was involved.

Minor outcomes were protracted symptoms and general well-being in abstinent patients, as well as side effects.

### Scale for Protracted Withdrawal Symptoms of Heroin Users

Protracted symptoms were examined and quantified by the Scale for Protracted Withdrawal Symptoms of Heroin Users. It is a Chinese questionnaire developed by Shi et al. (19) in 2009, and it evaluates the severity of protracted withdrawal symptoms in people using heroin. Withdrawal symptoms are assessed 2, 3, and 4 weeks after admission to a compulsory detoxification center. The scale consists of 19 items written in plain Mandarin, and five levels of rating (points ranging from 0 to 4). It includes four factors, namely physical symptoms, mood symptoms, symptoms of craving, and difficulty in sleeping. Each subscore is a simple add-up of points. The items covering physical symptoms (0–20 points) are “My heart beats rapidly”; “I feel unspeakable discomfort all over”; “‘I feel discomfort no matter where I place my hands and legs”; “I feel aches in muscles or joints”; “I feel out of strength”. The items exploring mood symptoms (0–16 points) include “I fidget about”; “I feel lonely”; “I do not feel interested in anything”; “I lose my temper over trivial matters”. The items investigating symptoms of craving (0–24 points) include “I want to take some heroin when I had poor sleep”; “Without the drug, life is way too long to endure”; “I always think about getting some heroin”; “I want to take some heroin when I get bored or upset”; “I want to take some heroin when I see people or things reminding me of heroin”. The items covering sleep problems (0–16 points) are “I feel I am lacking sleep”; “I have difficulty to fall asleep”; “I sleep lightly, I am easily woken up in the middle of the night”; “I wake up too early”. The Cronbach indices were 0.8127, 0.7950, 0.9041, and 0.8501, respectively. The correlations between factors were around 0.5240–0.8550. The product moment coefficients of correlation of the repeated measures were 0.8727, 06440, 0.7339, 0.8263, and 0.8943 for the scale and the four factors. During day 30 to 45 after withdrawal, the subscores of physical symptoms, unstable mood condition, craving, and sleep problems were 3.00 ± 3.51, 2.94 ± 2.82, 4.59 ± 5.60, and 4.80 ± 4.32, respectively (19).

### Short-Form 36

General health was assessed by the Short-Form 36, which was developed at RAND Corporation as part of the Medical Outcomes Study as a self-report, 36-item survey measuring health-related quality of life (20). Thirty-five items are used to construct eight sections: vitality, physical functioning, bodily pain, general health perceptions, and physical role functioning, emotional role functioning, social role functioning, and mental health. An additional item measures health transition. Raw scores are calculated as the sum of re-coded scale items and transformed to a 0–100 scale, with a score of 100 suggesting no disability. Two summary measures known as component scores are derived: the physical health component score and the mental health component score. All scales and the component scores are positively scored so that higher scores represent better health-related quality of life.

### Statistical Analysis

Quantitative data were presented as mean ± standard deviation. Kolmogorov–Smirnov test was applied to assess normality. Student t test was carried out for comparison of age between two groups. Mann–Whitney U test was used for quantitative parameters which do not have a normal distribution and chi-square test for comparison of categorical data. In abstainers, comparison of protracted symptoms and healthy conditions in buprenorphine-free and maintaining subjects was carried out by t test with false discovery rate (FDR) correction. *P* value or FDR < 0.05 was considered as statistically significant.

## Results

### Demographic Characteristics

Screening the previous medical history yielded a list of 271 patients who had used BMT in the Center. Among them, 110 patients were not able to be reached because the contact information was no longer valid; 88 patients refused to answer the call from the Center, 1 patient refused to give informed consent, and 72 patients (or their close relatives) were interviewed. Out of these 72 patients, 67 were male. They were 34.90 ± 6.85 years old, and their years of education were 9.47 ± 2.06. More than half of the patients were self-employed; only three had jobs as employees. The majority (45/72) of the patients were married or lived with a partner. The patients had begun using illicit drugs at the age of 24.57 ± 6.40. The main route of use was smoking and/or injecting.

### Main Outcome

**Figure 1:**
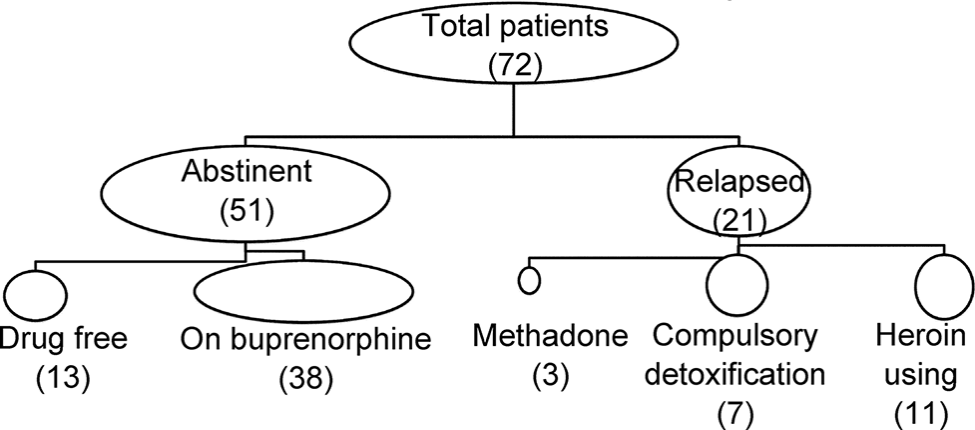
Main outcome of patients with minimal-dose buprenorphine maintenance. Out of a total number of 72 patients, 51 remained abstinent, and 21 were either transferred to compulsory treatment or returned to heroin use.

Patients who had finished inpatient detoxification started on BMT; the time of maintenance was about 33 ± 24 months (ranging from 6 to 108 months) prior to the survey. As shown in **Figure 1**, there were 51 patients who remained abstinent, out of which 13 subjects were free from any opioid including buprenorphine, and 38 patients were on buprenorphine. The remaining 21 patients were coded as relapsed, including 11 using heroin, 7 transferred to compulsory detoxification, and 3 transferred to MMT.

### Predictors of Worse Outcomes

As shown in **Table 1**, basic demographics and drug-use features were analyzed to find possible predictors of the primary outcome. We found relapsed patients were younger and had initiated heroin at younger age. They also had relatively fewer educational years. No difference was found in marital status, occupation, dose of heroin use, routes of heroin use, or initial dose of buprenorphine.

**Table 1:** Factors in relation to primary outcome (educational level and onset age of heroin use).

Groups	No. of subjects	Gender: Male	Age (years)	Educational years	Occupation	Marital status	Onset age of heroin use (years)	Heroin dose per day (g)	Main route of heroin use	Initial dose of buprenorphine (mg)
Abstainers	51	47	36.5 ± 6.5	9.9 ± 2.2	Self-employed: 28 Employee: 3 No job: 14	Single or divorced: 14 Married or with a partner: 31	25.8 ± 6.8	0.59 ± 0.59	Smoke/sniff: 19 Smoke/sniff and inject: 3 Inject: 16 Oral (methadone): 3	1.3 ± 0.9
Relapsed subjects	21	20	31.7 ± 6.6	8.7 ± 1.6	Self-employed: 10 Employee: 0 No job: 11	Single or divorced: 7 Married or with a partner: 14	22.0 ± 4.7	0.92 ± 0.75	Smoke/sniff: 8 Smoke/sniff and inject: 1 Inject: 12 Oral (methadone): 0	1.1 ± 0.7
*P* value	–	0.64	0.008	0.036	0.16	0.86	0.016	0.95	0.41	0.16

Educational years, age, and onset age of heroin use are different between abstinent subjects and relapsed ones. P values were calculated by independent two-sample t test (ages, educational years, onset age of heroin use), Manny–Whitney U test (heroin dose per day, initial dose of buprenorphine), and chi-square test.

### Reported Side Effects

As listed in **Table 2**, loss of weight is the most common side effect (7/38) noticed by patients. Weight loss was between 10 and 20 kg, and it was not associated with loss of appetite. This seemingly long-term effect has not been mentioned in previous medical records but was raised by some patients at the last follow-up. The second most common side effect was constipation, which was self-reported in four patients, but mostly described as tolerable. Other symptoms mentioned were loss of appetite, sweating, and decline of memory.

**Table 2:** Side effects reported in 38 patients during their use of buprenorphine. Intravenous use was present in one patient.

Patient ID	Age	Initial dose (mg/day)	Current dose (mg/day)	Months of BMT	Side effect(s)
No. 18	46–50	1.25	0.83	14	Loss of weight, decline of memory
No. 19	41–45	3.3	0.83	22	Loss of weight
No. 31	26–30	1.5	0.5	21	Loss of weight
No. 32	31–35	1.5	1.5	9	Loss of weight
No. 39	26–30	1.5	1.5	36	Loss of weight, constipation
No. 49	31–35	3	1.75	77	Loss of weight
No.21	41–45	1.25	0.75	105	Constipation
No. 36	31–35	0.5	0.5	26	Constipation, loss of appetite
No. 40	31–35	2.75	0.58	49	Constipation
No. 30	41–45	1.5	3	10	Sweating
No. 50	31–35	2	2.5	22 months under BMT + 14 months abuse	Intravenous use

BMT, buprenorphine maintenance treatment.

**Figure 2:**
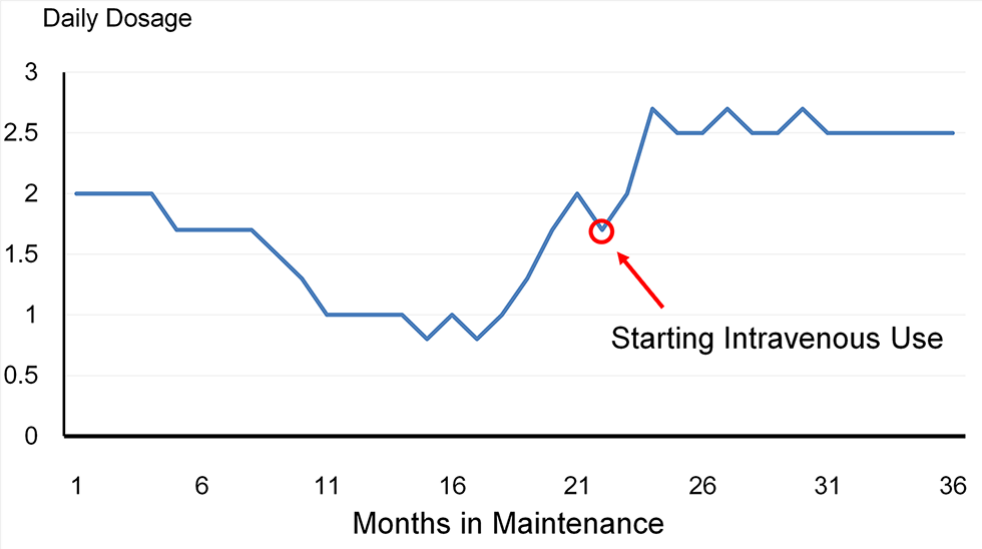
The trajectory of the patient who used buprenorphine intravenously. The dosage was reduced over a period of 16 months, and then increased gradually to 2 mg/day in the 22nd month. The patient then started using buprenorphine powder intravenously for about 14 months until his second hospitalization.

Buprenorphine abuse was noted in one patient. He used sublingual medication intravenously after 22 months of buprenorphine maintenance which he continued for 14 months until his second hospitalization in June 2016 (**Figure 2**).

### Trajectory of BMT

In the abstinent patients, 13 were currently opioid-free. These patients had started buprenorphine 37 ± 15 months (median 41 months, ranging 8–53 months) before the last follow-up interview, at a dose of 0.95 ± 0.42 mg/day (median 0.83 mg/day, ranging from 0.3 to 1.75 mg/day). They had gradually decreased the dose and stopped the medication after 11 ± 8.7 months (median 12 months, ranging from 3 to 25 months). They had been in an opioid-free state for 26 ± 14 months (median 29 months, ranging from 5 to 53 months).

**Figure 3:**
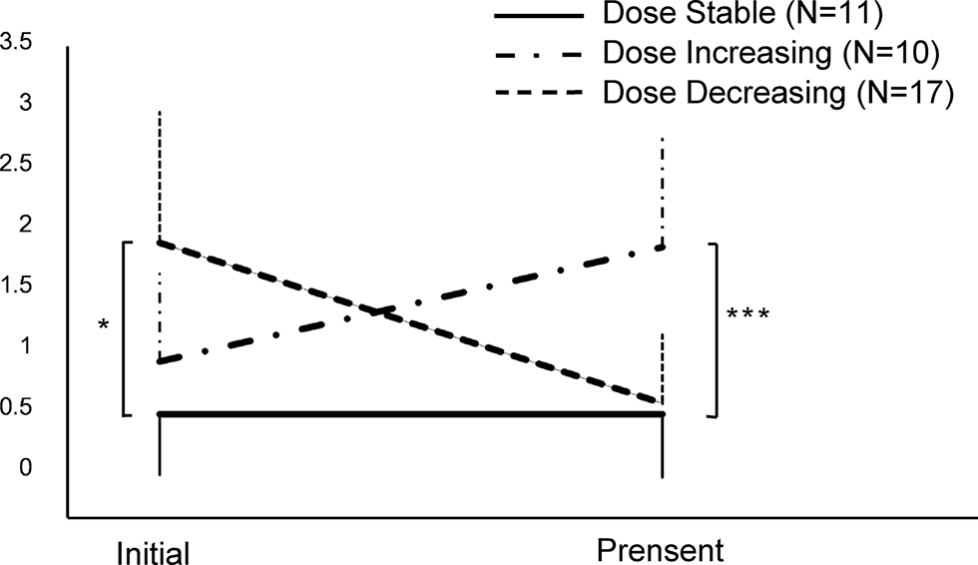
The changes of dose in patients currently maintained on buprenorphine (N = 38). *p < 0.05, ***p < 0.001, by one-way ANOVA.

The remaining 38 patients were still maintained on buprenorphine. They had been on the medication for 31 ± 27.7 months (median 26 months, ranging from 3 to 108 months). Their initial doses were higher than the initial doses of the 13 drug-free abstainers (*p* = 0.011), at an average of 1.48 ± 0.95 mg/day (median 1.3 mg/day, ranging from 0.3 to 3.5 mg/day). Their current doses were 1.2 ± 0.8 mg/day (median 0.8, ranging from 0.2 to 3.2 mg/day). The trajectory of dose changes varied in individual patients. Generally, 17 subjects could be categorized as “dose decreasing,” whereas 11 and 10 subjects could be categorized as “dose stable” and “dose increasing,” respectively. As depicted in **Figure 3**, the initial doses were higher in dose decreasing patients than others (p = 0.016), and the current doses were higher in dose increasing subjects (*p* < 0.001).

### Health Condition of Abstinent Patients

Protracted withdrawal symptoms and general health were investigated in patients who were abstinent from heroin (**Table 3**). In general, abstinent patients had only few protracted symptoms, including craving. Their scores on physical symptoms, unstable mood condition, craving, and sleeping disorders were 1.00 ± 1.50, 0.66 ± 1.24, 0.70 ± 1.67, and 2.32 ± 3.05, respectively. Their health condition was relatively good as examined by Short-Form 36. Opioid-free abstainers were better off than buprenorphine maintainers in physical functions. They had reported less physical protracted symptoms and had a higher physical health score in the health questionnaire.

**Table 3:** Protracted withdrawal symptoms and health condition for abstainers on or without buprenorphine.

	Drug-free (13)	On buprenorphine (38)	*P* value	FDR
Protracted symptoms				
**Physical symptoms**	**0.23 ± 0.60**	**1.27 ± 1.63**	**0.008**	**0.02**
Unstable mood condition	0.08 ± 0.28	0.86 ± 1.38	0.025	0.06
Craving	0.00 ± 0.00	0.95 ± 1.88	0.039	0.06
Sleep problems	1.15 ± 2.15	2.83 ± 3.24	0.084	0.06
General health				
General health perceptions	77.2 ± 11.5	63.8 ± 21.0	0.030	0.06
**Physical functioning**	**99.6 ± 1.4**	**94.3 ± 12.8**	**0.004**	**0.02**
Physical role functioning	100 ± 0	77.7 ± 34.8	0.015	0.05
Emotional role functioning	100 ± 0	82.0 ± 30.0	0.029	0.06
Social role functioning	90.6 ± 4.2	84.2 ± 15.4	0.191	0.19
Bodily pain	96.8 ± 8.1	86.9 ± 17.1	0.048	0.06
Vitality	66.9 ± 8.8	60.4 ± 12.2	0.051	0.06
Mental health	77.2 ± 8.2	69.7 ± 13.0	0.049	0.06
**Physical health score**	**56.9 ± 1.9**	**52.5 ± 6.2**	**0.003**	**0.02**
Mental health score	51.5 ± 3.0	47.6 ± 7.2	0.050	0.06

FDR, false discovery rate.

P values were calculated by independent two-sample Manny–Whitney U test. Higher scores in protracted withdrawal symptoms indicate more severe symptoms; higher scores in general health indicate better health condition.

The bolded texts are those with FDR < 0.05.

Differences in health status between drug-free subjects and those maintaining on BMT could not be explained by their features of heroin use history or the initial dosage of BMT. There was no significant difference in protracted symptoms and health conditions among those with increasing, stable, or decreasing BMT dosages.

## Discussion

Our findings suggest that using a smaller dose of buprenorphine for substitute treatment achieves substantial effectiveness. The primary data show that adding a prior procedure of detoxification to BMT can reduce buprenorphine doses and maintain heroin users in a satisfying health condition. Dosages under ≤ 2 mg/day buprenorphine were effective, as 51 out of 72 patients had managed to remain abstinent, and had experienced little craving or protracted symptoms.

Patients with complete follow-up data reported low protracted symptoms, including physical symptoms, unstable mood condition, craving, and sleeping disorder. Although direct comparison is not available, the protracted scores in these patients seemed much lower than those reported in detoxification center during day 30 to 45 after withdrawal (19). It suggests that the dosages of BMT were sufficient for these patients, and the detoxification before BMT introduction was successful in reducing patients’ need for an opioid. An interesting finding is that drug-free abstainers reported better physical and psychological health than those maintaining on buprenorphine, which is consistent with the report that patients with long-term naltrexone maintenance manifest an improvement in depression over time (21). Such a phenomenon might be explained by the physical and psychological homeostasis over time influenced by both pharmacological intervention and improvement of socioeconomic status.

With accumulating clinical data on relapse prevention, the focus of treatment for heroin use disorder has transferred from strict management and requirement of thorough detoxification to more client-centered and pragmatic methods. This is reflected in approaches that focus on retention of patients in treatment (22), in changing attitudes toward abstinence-oriented policies (23), and in development of heroin-injection programs for refractory patients (24). On the other hand, it can be argued that a more laissez-faire attitude could increase the risk of opioid overdose. Habituation to larger dosages of opioids might increase an individual’s sensitivity to pain (25), increase tolerance to pain killers and anesthetic medications (25; 26), exacerbate erectile dysfunction (27, 28), confine patients to lifetime medication, and increase incidence of death (29).

The approach of minimal-dosage BMT is a way to provide a client-centered treatment that encourages and fulfills many patients’ wish to achieve opioid abstinence. We believe dependency on opioids, both physical and psychological, can be overcome gradually. Detoxification provided a preparation for subsequent treatment and many patients’ goal of total abstinence. Protracted symptoms and reward deficiency can last many months (30). Previous detoxification and antagonist therapies failed to yield prolonged protection in the majority (2, 31) probably because the patients could not tolerate these protracted effects. It appears that the detoxification procedure in this study largely reduced an individual’s tolerance to opioids. The combination of a prior detoxification regime and subsequent maintenance therapy can therefore be viewed as a titration method to ameliorate an individual’s protracted symptoms and enable them to achieve their ultimate goal of full abstinence.

Several cultural factors should be considered in the application of this method. In China, the regulation of drugs is strict and has been tightened again in recent years. Stigma and public fear toward illicit drug use are highly relevant. Therefore, patients may have a stronger motivation or feel pressured to achieve full abstinence rather than continue maintenance treatment. Chinese family structures may be considered a protective factor. Traditionally, close parental–offspring support can last for a lifetime (32), which possibly helps to compensate for many patient’s precarious socioeconomic status. Thirdly, the patients in this study had comparable educational levels to the general population in China, as reported by the national population sampling in 2015 (33). Current abstinence is associated with higher education, older age, and later onset of heroin use. Lastly, the individual economic burden of having to pay for medication may contribute to an individual’s motivation to achieve abstinence and to reduce the dosage of buprenorphine. Nonetheless, the strategy suggested in this study can be applied in patients with a high motivation for drug abstinence.

Misuse of buprenorphine intravenously was seen in one patient. This patient’s dosage was increased after an initial decrease, which may suggest that maintaining him on a low dose was not successful, which led to a return to illicit drug use. He was hospitalized again for further treatment. This case suggests caution when reducing or increasing dosages and the necessity to explore why the need for opioids varies over time.

There are a few limitations in this study.

Firstly, the outcomes were self-reported and not cross-verified by other objective measures. In such cases, it is likely patients minimized the problems and overstated their health condition. Secondly, the proportion of patients willing to be interviewed was small. The contact information was often not valid, and a number of patients declined to answer the call from the clinic, reflecting the stigma and the psychological pain the disorder carries. This might suggest a risk of bias due to selective reporting.

Thirdly, only patients who had been maintained on BMT for at least 1 month participated in the interview. This could have increased selection bias, as patients who needed higher dosages may have dropped out prematurely in the adapting period (although it was not a requirement to stay on a low dose to continue treatment). The 1-month period was, however, necessary to ensure that a therapeutic relationship between patient and clinician could be established, that patients were able to adhere to the treatment regime by attending the clinic regularly, and that they could financially afford the use of BMT.

This study also did not record the patients’ comorbidities, which may substantially affect individual dosages and outcomes (10). The clinic does not provide treatment on comorbidities, as patients are usually referred to their general practitioner when health problems arise, so data were not available in a format which could have been used for this study. In the final interview, we assessed the general health in abstinent patients, which suggested absence of severe comorbidities that may impair the social function, but there is no solid information available about comorbidities in patients who relapsed or refused to participate.

Last but not least, no comparison was made between patients on low-dose buprenorphine and patients treated with a standard BMT procedure, so it is not possible to claim that low-dose BMT has a comparable effect to conventional treatment. One of the benefits of higher-dose BMT is better retention outcome, as shown in both clinical trials (5) and naturalistic settings (34). In a low-dose setting, patients may drop out early and return to illicit opioid use, which cases we failed to examine in this study. A low-dose treatment with a prior detoxification procedure may, however, enable patients to become drug-free, as shown in our previous study using detoxification technique only (18).

In conclusion, this study shows that there can be an alternative to the current treatment of opioid use disorders: BMT with a dose of less than 2 mg per day post-detoxification causes only little protracted symptoms, improves general health, and offers a buffering and strategy for patients who want to be free from opioids. Further studies comparing low-dose BMT with conventional BMT should be carried out in China and worldwide.

## Data Availability Statement

The datasets generated for this study are available on request to the corresponding author.

## Ethics Statement

The studies involving human participants were reviewed and approved by Ethics Committee of Human Studies of Ningbo Addiction Research and Treatment Center. Written informed consent for participation was not required for this study in accordance with the national legislation and the institutional requirements.

## Author Contributions

WS organized the survey and wrote the manuscript. QW, WP, JWZ, WY, and QH participated in the data collection and contributed in discussion. JBZ provided treatment details and contributed in discussion. DC participated in writing the manuscript and contributed in discussion. WZ designed the study, participated in writing the manuscript, and organized discussion.

## Funding

This research was supported by the National Nature Science Foundation of China (81671321) and National Basic Research Program of China (2015CB553504), Natural Science Foundation of Ningbo Municipality (2017A610224).

## Conflict of Interest

WS, QW, JZ, and WZ are working in the institute that involves voluntary addiction treatment in Ningbo. None of the authors declare connection with tobacco, alcohol, cannabis, pharmaceutical, or gaming industries.

The remaining authors declare that the research was conducted in the absence of any commercial or financial relationships that could be construed as a potential conflict of interest.
